# The Art of Intertwining Life and Work

**DOI:** 10.3201/eid2009.AC2009

**Published:** 2014-09

**Authors:** Byron Breedlove, Komatra Chuengsatiansup

**Affiliations:** Centers for Disease Control and Prevention, Atlanta, Georgia, USA (B. Breedlove);; Ministry of Public Health, Nonthaburi, Thailand (K. Chuengsatiansup)

**Keywords:** art science connection, emerging infectious diseases, pathogens, microbes, mixed farming, art and medicine, Pornchai Jaima, Integrated Farming, The Art of Intertwining Life and Work, about the cover

**Figure:**
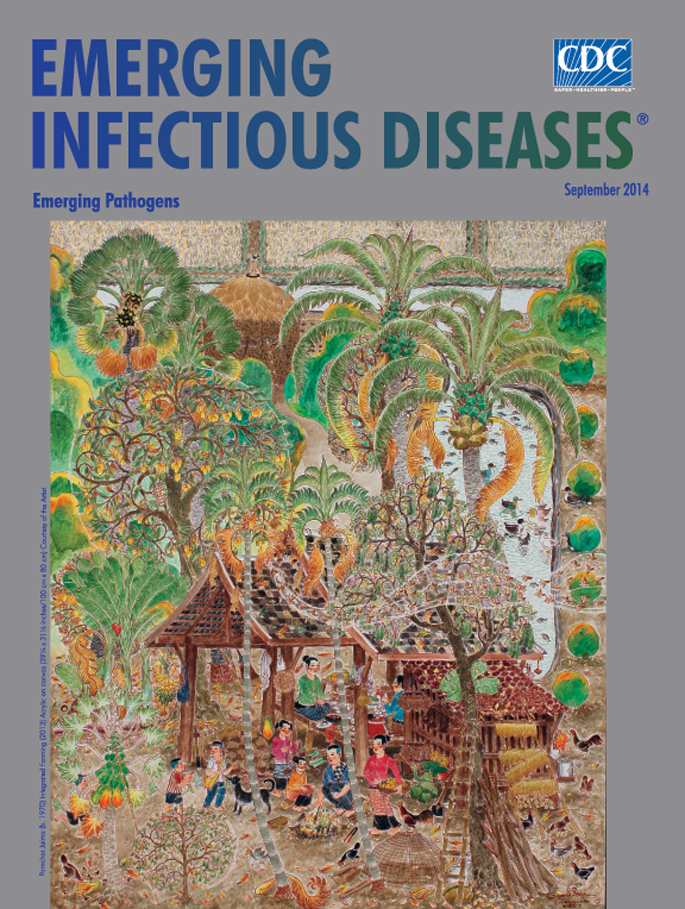
**Pornchai Jaima (b. 1970) Integrated Farming (2013) Acrylic and pastel on canvas (39 1/4 × 31 1/2 inches/100 cm × 80 cm)** Courtesy of the Artist

Pornchai Jaima grew up in rural northern Thailand, village life, work, and worship were all intertwined. His parents were wood carvers at the local Buddhist temple, where at age 10 he began studying traditional northern Thai Buddhist scriptures. Upon entering the sixth school grade at age 12, Jaima became a Buddhist novice for three years of study and meditation.

After completing high school, Jaima attended a local fine arts college in Chiang Mai and began painting murals. Inspired after meeting the artist Chalermchai, he continued his studies at Silpakorn University. Before taking the admission examination, Jaima vowed to Khruba Srivichai, a Buddhist master of northern Thailand, that if he passed, he would return home to build temples.

At the university, he began painting in the traditional northern Thai mural style, focusing on work, leisure, and customs of everyday village life in northern Thailand. Jaima has exhibited his work in the United States, France, the People’s Republic of China, and Japan. He has won many awards, including the prestigious Silpathorn Award in Visual Arts. He regularly paints murals for temples and is building a meditation center that he will decorate with his own art for the community.

*Integrated Farming, *this month’s cover image, reveals a lush tropical setting. Colorfully dressed villagers appear at a homestead, where there is space both for agricultural production and family activities. Pots, bowls, and cups cover the table. A woman carries a basket of mangoes, and flat baskets of peppers are drying on the lower roof. Bananas hang from the corner of the pavilion; papaya and mango trees are loaded with gold and green fruits. The shelves and tables are piled with foods.

As man tends a small fire, roasting glutinous rice in bamboo joints, 2 boys pretend to ride “horses” made from banana leaves and are greeted by the village dog. A girl plays with a walking toy made from coconut shells.

Two areca palms, laden with betel nuts, jut point toward the canopy of tree tops where a riot of shapes and textures fans out like fireworks. Smoothly worn paths lead to a hut visible through the trees. Rice paddies glimpsed at the top of the painting reveal that the homestead is more extensive than first thought. Tools used for farming, harvesting, and cleaning stand idle as the villagers take time to prepare and enjoy a meal from the efforts of their own labors.

Hues of green, gold, and brown are the predominant colors. Jaima uses very little blue in this painting—there is not a glimpse of the sky. In keeping with the traditional painting technique of two-dimensional depiction, there are almost no shadows or reflections other than those under the ducks on the pale blue-gray water. A flock of birds swoops across the middle of the painting, disappearing into nests woven from dried leaves.

Jaima pays extraordinary attention to details in this idyllic folk art scene of everyday life. Joseph Campbell observed that in Buddhist art in Far Eastern countries the accent is often on “the rich garden of this beautiful world itself, where things comfortable in their places may be recognized as themselves divine in their very idiosyncrasies.”

In this somewhat utopian depiction of traditional village life, the integration of agriculture and aquaculture offers an economically and environmentally viable means of sustenance. Fruit trees, livestock, vegetables, rice, fish, and water sustain the people. The various components of this system of mixed farming, in turn, support each another. The household poultry eat insects, fallen fruit, and weeds and provide fertilizer for the soil. Fish feed on vegetable matter and help fertilize the rice paddies. Bamboo and other trees provide fuel, materials, shelter, and tools.

Humans in rural settings have lived in close proximity with domestic and wild animals for millennia. These self-sufficient villages have sustained generations of people but may be susceptible to climate change and other threats.

The demand for wood and wood products, land for agricultural and living space, and minerals and other natural resources threatens the ecosystems across Southeast Asia, which also increases the potential exposure to emerging pathogens. Many emerging human, domestic animal, and wildlife diseases may infect multiple hosts.

While monocropping and modern agroindustrial practices raise concerns of emerging infectious diseases, remote and rural villages where mixed farming sustains people can become crucibles for emerging microbes and pathogens. One hopes that Jaima’s wonderful paintings of rural Thai life are not someday merely retrospectives that show us a lost traditional way of life.
